# The Delay in Issuing This Number

**Published:** 1893-01

**Authors:** 


### HOMŒOPATHIC PHYSICIAN,

A MONTHLY JOURNAL OF

HOMŒOPATHIC MATERIA MEDICA AND CLINICAL MEDICINE.

"If our school ever gives up the strict inductive method of Hahnemann, we are lost, and deserve only to be mentioned as a caricature in the history of medicine."—CONSTANTINE HERING.

Vol. XIII. JANUARY, 1893. No. 1.

### EDITORIAL.

THE DELAY IN ISSUING THIS NUMBER.—In wishing the subscribers to THE HOMŒOPATHIC PHYSICIAN a Happy New Year, the editor must also apologize for the late appearance of both the December and January numbers. This delay was owing, in the first place, to the work of getting out the index for the year. To the inexperienced in such matters, a perusal of the index will not impress them with the magnitude of the task. We can assure them, however, that it is really immense. This work has delayed the December number and consumed the time that should have been given to the number for the present month.

It is also to be regretted that a great deal of valuable matter had to be crowded out this month. It would have been a pleasant duty to the editor to have given much more of the proceedings of the Internationals. It was not possible to do so, however, within the limits of the number of pages which our financial resources will allow. Therefore these papers have been postponed. The Preface to *The Organon*, on page 46, is published by request of Dr. Fincke, as an answer to Dr. Wesselhœft's attack in *The Homœopathic Recorder*.

